# Symptoms of Dry Eye Disease and Personality Traits

**DOI:** 10.1371/journal.pone.0166838

**Published:** 2016-11-18

**Authors:** Sho Ichinohe, Tsutomu Igarashi, Daisuke Nakajima, Masafumi Ono, Hiroshi Takahashi

**Affiliations:** Department of Ophthalmology, Nippon Medical School, 1-1-5 Sendagi, Bunkyo-ku, Tokyo 113–8602, Japan; Save Sight Institute, AUSTRALIA

## Abstract

The essential targets of dry eye disease (DED) treatments include both objective signs and subjective symptoms. However, due to the numerous subjective symptoms, it is understandable why little association has been found between the signs and symptoms. Although psychological influences on the subjective symptoms have been reported, little is known about the influence of personality traits. The present study analyzed the relationship between the signs/symptoms of DED and the personality traits of patients using a cross-sectional design. We examined 56 DED patients (mean age; 62.4 ± 12.9, range 34–85 years) visiting the outpatient clinic of the Department of Ophthalmology at the Nippon Medical School Hospital in Tokyo, Japan. Objective signs evaluated included the Schirmer I test, tear breakup time (BUT), fluorescein and lissamine green staining, and tear osmolality. Subjective symptoms were assessed by the Ocular Surface Disease Index (OSDI) and Dry Eye-Related Quality-of-Life Score (DEQS) questionnaires. For personality traits, the Big Five personality traits model analysis was used. Correlations between the objective signs, subjective symptoms, and personality traits were analyzed. A significant correlation was found between the neuroticism in the Big Five Personality Inventory and the symptoms assessed by the DEQS (r = -0.35, p < 0.01), and the OSDI (r = -0.28, p < 0.05). There was no significant correlation observed between the signs and the symptoms, or between the signs and any personality traits. The results of our current study suggest that the personality of the patient, which appears to be the basis of various psychological factors, can have some impact on the subjective symptoms. This may be one of the reasons why there has been little association noted between the signs and symptoms of DED.

## Introduction

In the 2007 International Dry Eye Work Shop report, dry eye disease (DED) was classified as a multifactorial disease [1] that had numerous subjective symptoms such as pain, dryness, grittiness, itchiness, redness, burning, foreign body sensation, light sensitivity, or fatigue. However, there has been limited objective assessment of the signs associated with DED, which include the Schirmer I test, tear film stability, and fluorescein staining. As a result, there has been little reported on any associations between the DED signs and symptoms [2–4]. On the other hand, multiple studies have examined the effects of the psychological influences including depression, anxiety, or feelings of subjective happiness on the subjective symptoms, and have reported finding a positive correlation [5–11]. In addition, pain sensitivity may also play a role in the DED symptoms. It has been reported that high pain sensitivity and low pain tolerance were associated with symptoms of DED [12], and that DED symptoms were more closely aligned to non-ocular pain than to tear film parameters [13]. These previous findings suggest that conditions other than the ocular status may have an impact on the subjective symptoms of DED. Considering a patient's psychological status, it is well known that personality traits do play an important role [14]. However, as far as we know, there have yet to be any studies that have reported finding any relationship between the personality traits and the subjective symptoms. Therefore, our current study investigated potential correlations between objective signs, subjective symptoms, and personality traits in patients with DED.

## Methods

### Study Design

We conducted a cross-sectional study of the patients seen at the Dry Eye Clinic at Nippon Medical School Hospital between 2013 and 2015. DED diagnosis was based on the standard dry eye diagnostic criteria currently used in Japan [13]. All patients diagnosed as DED had undergone DED medical treatments of at least three months or more. The patients who were using psychotropic medications were excluded. The research protocol followed the tenets of the Declaration of Helsinki and was approved by the Institutional Review Board of the Nippon Medical School Hospital. All participants provided their written informed consent prior to their participation in the study.

The main purpose of the current study was to determine whether a significant correlation exists between subjective symptoms of DED and some personality traits in DED patients. Thus, a non-DED control was not configured. In addition, the current study analyzed all of the available data obtained from the DED patients who visited the hospital during the study period. Therefore, it was not necessary to determine the sample size required for sufficient statistical power.

### Outcome Measures

The outcome measures of the current study consisted of objective parameters, symptom assessment, and personality evaluation.

### Diagnosis of DED

Patients enrolled in the study met all of the Japanese DED diagnostic criteria, including (1) presence of dry eye symptoms, (2) qualitative or quantitative disturbance of the tear film, and (3) ocular surface epithelial staining. The qualitative or quantitative disturbance of the tear film was defined as a positive result when the Schirmer I test was ≤ 5 mm or non-invasive BUT was ≤ 5 sec. The ocular surface epithelial staining was determined by positive vital staining using fluorescein or lissamine green. The degree of staining in the temporal and nasal conjunctiva and cornea, which was divided into three parallel sections, was recorded and quantified on a score of 0 to 3. Therefore, the maximum score (fluorescein staining score or lissamine green staining score) for one eye would be 9. If either type of staining was positive, the ocular surface was considered abnormal. Patients with subjective symptoms, in whom signs regarding tear dynamics and ocular surface were abnormal, were considered to have definitive DED.

All examinations were performed in the following order. First, tear osmolality was measured by Tearlab (Tearlab, San Diego) according to the Tearlab manual. Ocular surface damages were then assessed by fluorescein staining and lissamine green staining, and non-invasive BUT was measured. Finally, the aqueous tear volume was measured by the Schirmer I test. Both eyes were tested, with the data of both eyes used to calculate the parameters in the staining examination. Eyes having the worse value were used in the other examinations.

### Dry Eye Symptoms Questionnaires

Ocular Surface Disease Index (OSDI) [4] and Dry Eye-Related Quality-of-Life Score (DEQS) [15] questionnaires were used to assess the subjective symptoms. OSDI, which is one of the most commonly used questionnaires for DED, includes twelve questions regarding the visual functions, social activity and environmental influences. The DEQS, which has recently been proposed for use in Japan, consists of fifteen questions designed to examine the visual symptoms and general functions, including emotional conditions. All questionnaires were presented in Japanese.

### Personality Evaluation

After the subjective symptoms were assessed by OSDI and DEQS, the personality evaluation was performed. To evaluate the personality of the patients, our current study used the Japanese version of the Big Five Personality Inventory [16]. The five factor model, which is also known as the Big Five personality traits, was designed on the basis of the concept that one’s personality is composed of five factors [17]. The five factors, which are defined below, include neuroticism, openness, conscientiousness, extraversion and agreeableness.

Neuroticism refers to an emotional instability. Individuals with a high neuroticism are emotionally reactive and have an emotional response to events that would not affect most people. In the current study, we used the Japanese version of the Big Five Personality Inventory, in which a higher score is defined as being emotionally stable. Thus, individuals with low neuroticism scores are prone to having negative emotionality and being more sensitive to danger.

Openness refers to being open to new experiences. Individuals with a high openness will exhibit curiosity when approaching new things and be willing to entertain novel ideas and unconventional values.

Conscientiousness refers to the personality trait of being thorough, careful, or vigilant. Individuals with a high conscientiousness are identified as being strong and hardworking and are more easily intimidated.

Extraversion refers to the quantity and intensity of positive emotional adjustment, interpersonal interactions, and activity levels. Individuals with high extraversion are identified as individuals who tend to be sociable, active, optimistic, and able to appreciate experiences for their own sake.

Agreeableness refers to being compliant, trusting, empathic, sympathetic, friendly and exhibiting a cooperative nature. Individuals with high agreeableness are identified as individuals who build a friendly cooperative relationship with others and exhibit both sympathy and compassion.

The Big Five Personality Inventory questionnaire consists of 70 questions. After distribution, all of the participants were requested to complete this questionnaire. All analyses were conducted in accordance with the manual of the Big Five Personality Inventory.

### Statistical Analyses

Associations between the objective signs and the subjective symptoms, between the objective signs and the personality traits, and between the subjective symptoms and the personality traits were analyzed by Spearman’s correlations. In addition, multiple linear regression analysis was performed with the signs/symptoms parameters as the objective variables, and personality traits, age, and gender as the explanatory variables. All statistical analyses were done using SPSS software (IBM SPSS Statistics Version 21, Japan IBM, Tokyo, Japan).

## Results

### Patients Cohort Profile

The number of the DED patients enrolled in the study was 56. The mean age of the patients was 62.4 ± 12.93, and there was a male to female ratio of 3:53. The mean period of DED was 57 months ± 41 months. All patients were using lubricating eye drops such as artificial tears, and punctal plugs were inserted in 24 patients. None of the patients were using contact lenses. Co-morbidities included hypertension (10), gynecologic diseases (5), diabetes (2), asthma (1), allergy to pollen (1), and autoimmune diseases such as Sjögren syndrome (16), rheumatoid arthritis (7), systemic lupus erythematosus (2), dermatomyositis (1), and scleroderma (1).

### Symptoms and Signs

There was no correlation between the objective signs and the subjective symptoms (OSDI and DEQS) (Table 1).

**Table 1:** Objective signs and symptom scores (DEQS and OSDI).

Objective evaluations	mean ± SD	DEQS		OSDI	
		r	p-value	r	p-value
**Fluorescein staining score**	**2.36 ± 2.37**	**-0.076**	**0.573**	**-0.005**	**0.968**
**Lissamine green staining score**	**1.65 ± 1.75**	**-0.079**	**0.561**	**-0.043**	**0.755**
**BUT (sec)**	**2.80 ± 1.70**	**0.019**	**0.89**	**0.021**	**0.878**
**Schirmer I test** [12]	**7.32 ± 7.01**	**-0.019**	**0.888**	**-0.151**	**0.27**
**Tear osmolarity (mOsm/L)**	**286.51 ± 9.63**	**0.097**	**0.54**	**0.142**	**0.371**

No significant correlation is observed between any of the signs and symptom scores.

r; Spearman’s correlation coefficient.

### Signs and Personality Traits

**Fig 1:**
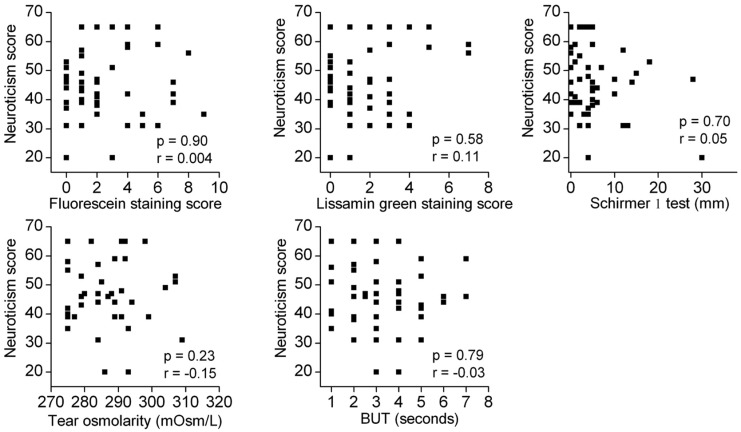
Scatter plots showing the correlations between the objective dry eye signs and the neuroticism score. The objective signs examined were the fluorescein staining score, lissamine green staining score, Schirmer I test, tear osmolarity, and the BUT. No significant correlations were observed for any of the combinations. Similarly, there were no other personality traits that exhibited any significant correlations with the objective signs (Data not shown). r; Spearman’s correlation coefficient.

There was no correlation between the objective signs and any of the personality traits (Fig 1).

### Symptoms and Personality Traits

**Fig 2:**
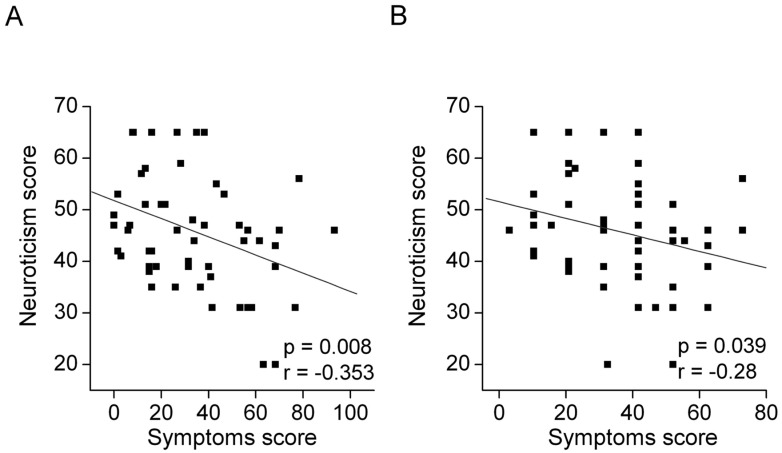
Correlations between the subjective dry eye symptoms and the neuroticism score in the scatter plots. A) DEQS and Neuroticism. Individuals with a lower neuroticism score (more nervous) exhibited a higher DEQS score (more symptoms). There is a significant correlation between the neuroticism score and the DEQS score (r = -0.353, p = 0.008). r; Spearman’s correlation coefficient. The gray line indicates the regression line. B) OSDI and Neuroticism. Individuals with a lower neuroticism score (more nervous) exhibited a higher OSDI score (more symptoms). There is a significant correlation between the neuroticism score and the OSDI score (r = -0.28, p = 0.039). r; Spearman’s correlation coefficient. The gray line indicates the regression line.

There was a strong correlation between the DEQS score and OSDI score (r = 0.837, p < 0.0001). There was no correlation between the subjective symptoms (OSDI and DEQS) and the four personality traits that included openness, conscientiousness, extraversion and agreeableness. In contrast, there was a significant correlation between the neuroticism and the OSDI score (r = -0.28, p = 0.039) and the DEQS score (r = -0.353, p = 0.008) (Table 2). The lower the neuroticism score was, the worse the symptoms were found to be (Fig 2). In addition, DEQS exhibited a better correlation with neuroticism compared to the OSDI. When data for the16 Sjögren syndrome patients were extracted, the correlations between the neuroticism and the OSDI score and the DEQS score became more evident (OSDI: r = -0.486, p = 0.056, and DEQS: r = -0.627, p = 0.001).

**Table 2:** The Big Five personality traits and symptom scores (DEQS and OSDI).

	Mean of the score Low/High	Mean ± SD	DEQS		OSDI	
			r	p-value	r	p- value
**Openness**	**Conventional/** **Imaginative**	**44.25 ± 10.81**	**0.015**	**0.913**	**0.075**	**0.587**
**Conscientiousness**	**Negligent/** **Conscientious**	**45.81 ± 9.88**	**0.075**	**0.587**	**-0.026**	**0.848**
**Extraversion**	**Passive/** **Active**	**48.60 ± 10.66**	**-0.118**	**0.391**	**-0.112**	**0.414**
**Agreeableness**	**Suspicious/** **Trusting**	**49.78 ± 9.18**	**0.078**	**0.573**	**0.09**	**0.512**
**Neuroticism**	**Pessimistic/** **Optimistic**	**44.83 ± 11.93**	**-0.353**	**0.008**	**-0.28**	**0.039**

The Big Five personality traits consist of openness, conscientiousness, extraversion, agreeableness, and neuroticism. Only neuroticism shows a significant correlation to the DEQS (r = -0.353, p = 0.008), and OSDI (r = -0.28, p = 0.039). DEQS and OSDI also show a significant correlation (r = 0.837, p < 0.0001). r; Spearman’s correlation coefficient.

Multiple linear regression analysis also revealed a significant association of neuroticism with the DEQS score (p = 0.02, β = -0.301, R^2^ = 0.187).

## Discussion

Associations between the signs and symptoms in DED are rarely reported. Similar to other published reports [2–4], our current study also found there was no significant relationship between the signs and symptoms. The reasons responsible for this discrepancy may be due to the fact that while there are numerous subjective symptoms, there are only limited methods that can be used to assess objective signs in DED. However, this inability to find any associations could be changed if there is the emergence of a new objective measure that would make it possible to explain the etiology of a symptom. Alternatively, the possibility also exists that subjective symptoms could be influenced by either psychiatric or psychogenic factors of the patients. However, if this was the case, then eye care practitioners would never be able to explain the cause of these symptoms by simply using standard ophthalmic examinations. In fact, some investigators have reported finding a significant relationship between a patient's symptoms and their psychogenic status, such as depression, anxiety, or a subjective happiness feeling [5–11, 18], which suggests these relationships may need to be taken into consideration. In addition, sensitivity to pain has been reported to play a significant role in the severity of the DED complaints [12, 13], which also suggests importance of the non-ocular status of the patients with the DED symptoms.

In our current study, we demonstrated there was a significant correlation between the neuroticism and the symptom scores. Since it is well known that personality traits play an important role with regard to the psychological status [14], our current results are in agreement with the previous reports on the association between depression or anxiety and dry eye symptoms. Moreover, these results provide substantial supporting evidence for the empirical findings noted by eye care practitioners during their daily DED clinics. Since individuals with a high neuroticism are thought to be emotionally reactive and prone to react to events that would not affect most people, symptoms associated with DED that seem unreasonable could perhaps be explained by the individual’s personality trait, i.e., neuroticism. The observed correlation with neuroticism was much more evident when using the DEQS versus the OSDI. The reason for this is likely due to the fact that the DEQS but not the OSDI includes questions regarding emotional conditions.

One important viewpoint concerning the relationship between personality and disease is that one’s personality not only can affect the symptoms but also the status of the disease. The significant influence by personality traits on a disease has been widely investigated in both cancer and heart disease [19, 20]. Although the current study found there was no significant impact due to the personality traits on the DED objective measurements, the possibility does exist, e.g., personality traits could affect a patient's disease status through the habits of their daily routines, which in all likelihood are influenced by their personality. Further investigations into these speculations will need to be undertaken.

Alternatively, it is also known that the disease symptoms can affect the personality of a patient. In a previous study of glaucoma patients [21], the authors speculated that the decreased quality of life seen in the patients due to a decreased visual function and fear of blindness might have induced some of the characteristic personality traits that were observed in the subjects. In cases of life-threatening diseases such as cancer, it is very understandable that there would be an influence on the personality caused by fear or disturbance related to the disease [22]. While the subjective symptoms in DED are definitely not as serious when compared to those in life-threatening diseases or vision-threatening conditions, the numerous symptoms of DED, which include pain, dryness, grittiness, itchiness, redness, burning, foreign body sensation, light sensitivity, or fatigue, could very well affect the personality characteristics of the patients. Further studies that assess whether there is a relationship between personality traits and particular symptoms of DED will need to be undertaken in the future.

One other viewpoint states that there is a genetic influence on both the disease and on the personality traits. Since it has been reported that there are specific genes associated with personality traits [21, 23], it would be likely that some genetic backgrounds can simultaneously affect the personality traits and the pathology of diseases. In the current study, a stratified analysis on Sjögren syndrome patients revealed a more evident correlation between the syndromes and the neuroticism. It is intriguing that our results showed that there was a strong association found in the Sjögren syndrome cohort. While a genetic background influence on this association is unknown at the present time, further investigations that need to be undertaken may be able to answer this question in the future.

One of the limitations of the current study was the small size of the subjects that took part in the study, along with the fact that most of the patients were female. This may reflect the common gender ratio that has been reported for DED. However, since gender is an important factor in personality traits, a similar study in male patients will also need to be performed in the future.

In summary, we found a significant association between neuroticism and the DED symptoms. Our current findings suggest that the personality of the patient, which is the basis of various psychological factors, does have some impact on the subjective symptoms.

## References

[1] Report of the international dry eye workshop (DEWS). Ocul Surf. 2007;5(2): 75–107. Epub 107. 10.1016/S1542-0124(12)70080-0. 17508116

[2] Hua R, Yao K, Hu Y, Chen L. Discrepancy between subjectively reported symptoms and objectively measured clinical findings in dry eye: a population based analysis. BMJ open. 2014;4(8). 10.1136/bmjopen-2014-005296 25168038 PMC4156796

[3] Nichols KK, Nichols JJ, Mitchell GL. The lack of association between signs and symptoms in patients with dry eye disease. Cornea. 2004;23(8): 762–770. 10.1097/01.ico.0000133997.07144.9e 15502475

[4] Schiffman RM, Christianson MD, Jacobsen G, Hirsch JD, Reis BL. Reliability and validity of the ocular surface disease index. Arch Ophthalmol. 2000;118(5): 615–21. 10.1001/archopht.118.5.615 10815152

[5] van der Vaart R, Weaver MA, Lefebvre C, Davis RM. The association between dry eye disease and depression and anxiety in a large population-based study. Am J Ophthalmol. 2015;159(3): 470–474. 10.1016/j.ajo.2014.11.028 25461298 PMC4329250

[6] Na KS, Han K, Park YG, Na C, Joo CK. Depression, stress, quality of life, and dry eye disease in Korean women: a population-based study. Cornea. 2015;34(7): 733–738. 10.1097/ICO.0000000000000464 26002151

[7] Kawashima M, Uchino M, Yokoi N, Uchino Y, Dogru M, Komuro A, et al Associations between subjective happiness and dry eye disease: a new perspective from the Osaka study. PLoS One. 2015;10(4). 10.1371/journal.pone.0123299 25830665 PMC4382322

[8] Labbé A, Wang Y, Jie Y, Baudouin C, Jonas J, Xu L. Dry eye disease, dry eye symptoms and depression: the Beijing Eye Study. Br J Ophthalmol. 2013;23: 1399–1403. Epub 1403. 10.1136/bjophthalmol-2013-303838 24013959

[9] Hallak JA, Jassim S, Khanolkar V, Jain S. Symptom burden of patients with dry eye disease: a four domain analysis. PLoS One. 2013;8(12). 10.1371/journal.pone.0082805 24349365 PMC3862676

[10] Fernandez CA, Galor A, Arheart KL, Musselman DL, Venincasa VD, Florez HJ, et al Dry eye syndrome, posttraumatic stress disorder, and depression in an older male veteran population. Invest Ophthalmol Vis Sci. 2013;54: 3666–3674. 10.1167/iovs.13-11635 23633658

[11] Wen W, Wu Y, Chen Y, Gong L, Li M, Chen X, et al Dry eye disease in patients with depressive and anxiety disorders in Shanghai. Cornea. 2012;31(6): 686–692. 10.1097/ICO.0b013e3182261590 22382595

[12] Vehof J, Kozareva D, Hysi PG, Harris J, Nessa A, Williams FK, et al Relationship between dry eye symptoms and pain sensitivity. JAMA Ophthalmol. 2013;131(10): 1304–1308. 10.1001/jamaophthalmol.2013.4399 23907167

[13] Galor A, Felix ER, Feuer W, Shalabi N, Martin ER, Margolis TP, et al Dry eye symptoms align more closely to non-ocular conditions than to tear film parameters. Br J Ophthalmol. 2015;99(8): 1126–1129. 10.1136/bjophthalmol-2014-306481 25710726 PMC13539458

[14] Lautenschlager NT, Kurz AF, Loi S, Cramer B. Personality of mental health caregivers. Curr Opin Psychiatry. 2013;26(1): 97–101. 10.1097/YCO.0b013e32835997b3 23041793

[15] Sakane Y, Yamaguchi M, Yokoi N, Uchino M, Dogru M, Oishi T, et al Development and validation of the Dry Eye-Related Quality-of-Life Score questionnaire. JAMA Ophthalmol. 2013;131(10): 1331–1338. 10.1001/jamaophthalmol.2013.4503 23949096

[16] Onogi H. Correlative materials between three types of the five major factor personality test (in Japanese). Japan Society of Personality Psychology. 2004;12(2): 82–89. 10.2132/personality.12.82.

[17] Costa PT, McCrae RR. The NEO personality inventory manual. Odessa, FL: Psychological Assessment Resources 1985.

[18] Kim KW, Han SB, Han ER, Woo SJ, Lee JJ, Yoon JC, et al Association between depression and dry eye disease in an elderly population. Invest Ophthalmol Vis Sci. 2011;52: 7954–7958. 10.1167/iovs.11-8050 21896858

[19] Turhal NS, Demirhan S, Satici C, Cinar C, Kinar A. Personality traits in cancer patients. Asian Pac J Cancer Prev. 2013;14(8): 4515–4518. 10.7314/APJCP.2013.14.8.4515 24083694

[20] Schiffer AA, Denollet J, Widdershoven JW, Hendriks EH, Smith OR. Failure to consult for symptoms of heart failure in patients with a type-D personality. Heart. 2007;93(7): 814–818. 10.1136/hrt.2006.102822 17344329 PMC1994460

[21] Mabuchi F, Yoshimura K, Kashiwagi K, Shioe K, Kanba S, Iijima H, et al Personality assessment based on the five-factor model of personality structure in patients with primary open-angle glaucoma. Jpn J Ophthalmol. 2005;49(1): 31–35. 10.1007/s10384-004-0134-3 15692771

[22] Nakaya N. Effect of psychosocial factors on cancer risk and survival. J Epidemiol. 2014;24(1): 1–6. 10.2188/jea.JE20130124 24270060 PMC3872518

[23] Vedhara K, Gill S, Eldesouky L, Campbell BK, Arevalo JM, Ma J, et al Personality and gene expression: do individual differences exist in the leukocyte transcriptome. Psychoneuroendocrinology. 2015;52: 72–82. 10.1016/j.psyneuen.2014.10.028 25459894 PMC4297539

